# Altered endothelial dysfunction-related miRs in plasma from ME/CFS patients

**DOI:** 10.1038/s41598-021-89834-9

**Published:** 2021-05-19

**Authors:** J. Blauensteiner, R. Bertinat, L. E. León, M. Riederer, N. Sepúlveda, F. Westermeier

**Affiliations:** 1 Institute of Biomedical Science, Department of Health Studies, FH Joanneum University of Applied Sciences, Graz, Austria; 2 Centro de Microscopía Avanzada, CMA-BIO BIO, Facultad de Ciencias Biológica, Universidad de Concepción, Concepción, Chile; 3 Instituto de Ciencias Biomédicas, Facultad de Ciencias de La Salud, Universidad Autónoma de Chile, Santiago, Chile; 4 Department of Infection Biology, Faculty of Infectious and Tropical Diseases, London School of Hygiene and Tropical Medicine, London, UK; 5 CEAUL - Centro de Estatística e Aplicações da Universidade de Lisboa, Lisbon, Portugal; 6 Institute of Medical Immunology, Charité-Universitätsmedizin Berlin, Corporate Member of Freie Universität (FU) Berlin, Humboldt-Universität Zu Berlin and Berlin Institute of Health (BIH), Berlin, Germany; 7 Centro Integrativo de Biología y Química Aplicada (CIBQA), Universidad Bernardo O´Higgins, Santiago, Chile

**Keywords:** Biomarkers, Medical research

## Abstract

Myalgic encephalomyelitis/chronic fatigue syndrome (ME/CFS) is a complex disease characterized by unexplained debilitating fatigue. Although the etiology is unknown, evidence supports immunological abnormalities, such as persistent inflammation and immune-cell activation, in a subset of patients. Since the interplay between inflammation and vascular alterations is well-established in other diseases, endothelial dysfunction has emerged as another player in ME/CFS pathogenesis. Endothelial nitric oxide synthase (eNOS) generates nitric oxide (NO) that maintains endothelial homeostasis. eNOS is activated by silent information regulator 1 (Sirt1), an anti-inflammatory protein. Despite its relevance, no study has addressed the Sirt1/eNOS axis in ME/CFS. The interest in circulating microRNAs (miRs) as potential biomarkers in ME/CFS has increased in recent years. Accordingly, we analyze a set of miRs reported to modulate the Sirt1/eNOS axis using plasma from ME/CFS patients. Our results show that miR-21, miR-34a, miR-92a, miR-126, and miR-200c are jointly increased in ME/CFS patients compared to healthy controls. A similar finding was obtained when analyzing public miR data on peripheral blood mononuclear cells. Bioinformatics analysis shows that endothelial function-related signaling pathways are associated with these miRs, including oxidative stress and oxygen regulation. Interestingly, histone deacetylase 1, a protein responsible for epigenetic regulations, represented the most relevant node within the network. In conclusion, our study provides a basis to find endothelial dysfunction-related biomarkers and explore novel targets in ME/CFS.

## Introduction

Myalgic encephalomyelitis/chronic fatigue syndrome (ME/CFS) is a debilitating multisystem disease of unknown etiology, characterized by an unexplained persistent fatigue not improved by rest, unrefreshing sleep, cognitive impairment, post-exertional malaise, chronic pain, gastrointestinal symptoms, and orthostatic intolerance. Symptoms can persist for years, and most patients never return to their normal functioning before disease onset, which decreases their quality of life. Unfortunately, there is no single biomarker available for accurate clinical assessment of ME/CFS patients, and its diagnosis is based on the exclusion of other pathologies, leading to a high rate of under-diagnosed cases^1^. Therefore, advancing our understanding of ME/CFS is critical for the development of objective diagnostic biomarkers and specific treatments.

Despite the pathophysiological mechanism is still unclear, a growing body of evidence has been associating ME/CFS with immunological abnormalities and inflammation in a subset of patients^2,3^. Endothelial cells (ECs) are actively involved in the regulation of the immune system^4^ and endothelial dysfunction (ED) is linked to inflammation and oxidative stress^5^. By releasing nitric oxide (NO), ECs regulate the vascular tone to enable an adequate blood and oxygen supply to tissues throughout the body. Conversely, inadequate NO production is a major cause of ED^6^. Recently, two studies have reported ED in vivo in ME/CFS patients^7,8^, supporting previous evidence of vascular abnormalities^9^ and reinforcing the assumption that ED is another piece of this complex pathophysiological puzzle.

In healthy vessels, NO is generated from L-arginine by the endothelial isoform of nitric oxide synthase (eNOS), in response to several stimuli including shear stress during exercise. Accordingly, eNOS-derived NO is considered a crucial player in cardiovascular homeostasis^6^. Interestingly, silent information regulator 1 (Sirt1) reduces inflammation and oxidative stress^10^ and increases the production of NO by activating eNOS in ECs^11^. Despite, the biological relevance of the Sirt1/eNOS axis both in endothelial and immune homeostasis, there are no studies addressing potential alterations in ME/CFS.

Significant attention has recently focused on the general role of microRNAs (miRs) in the maintenance of endothelial function^12^. miRs are small non-coding RNAs that are altered in many diseases and, consequently, proposed as suitable biomarkers due to their potential to predict, diagnose, or monitor diseases^12^. In this line of thought, several miRs profiles have been reported in plasma^13,14^, extracellular vesicles^15^, and peripheral blood mononuclear cells (PBMCs)^16,17^ from ME/CFS patients. However, there are no studies focused on identifying whether endothelial dysfunction-related miRs are altered in ME/CFS.

In this work, we selected a set of five miRs (miR-21, miR-34a, miR-92a, miR-126, and miR-200c) previously reported to regulate endothelial function via the Sirt1/eNOS axis both in ECs^18–28^ and observational clinical studies^22,29–32^. We evaluated their expression by means of real-time PCR in plasma from mild/moderate (mm) and severely affected (sa) ME/CFS patients compared with healthy controls (HC), considering several statistical adjustments based on the clinical-related data. In order to further support our findings, we also analyzed their expression using available microarray data reported in PBMCs from patients with ME/CFS^17^. Finally, we integrated our experimental findings by performing bioinformatics analyses to predict biological processes and potential targets linked to the Sirt1/eNOS-related miRs in ME/CFS.

## Methods

### Samples and clinical data

This study was approved by two independent Research Ethics Committees at the Medical University of Graz, Austria (Reference number: 31–059 ex 18/19) and by the UCL Biobank Ethical Review Committee-Royal Free (B-ERC-RF) London NHS Foundation Trust (Reference number: EC2018.005). Samples were provided by the UK ME/CFS Biobank (UKMEB) in accordance with a material transfer agreement signed by the London School of Hygiene & Tropical Medicine (London, UK) and the FH JOANNEUM Gesellschaft mbH—University of Applied Sciences (Graz, Austria). All methods were performed in accordance with relevant guidelines and regulations.

All participants included in this study signed an informed consent form before their samples were stored at the UKMEB^33^. The samples included (n = 87) plasma (1 ml vial/participant) from mild/moderate (n = 28) and severely affected (n = 30) ME/CFS patients and age-matched healthy subjects (n = 29) were collected in EDTA blood-tubes by qualified UKMEB´s staff members. All samples were provided without patient information. Data associated with clinical assessments were released after the study was completed: pulse oximetry; diastolic and systolic pressure; height; waist circumference; weight; body fat (bioimpedance), body muscle (bioimpedance); body metabolic rate; pain and fatigue (analog scale), blood tests (hemoglobin; white blood cell count; platelets; red blood cells; hematocrit; neutrophils, lymphocytes; monocytes; eosinophils; creatinine; creatine phosphokinase (CPK); C-reactive protein (CRP), questionnaires (symptoms experienced), and SF-36 Health Survey (SF-36v2). At enrolment, all participants ages ranged from 19 to 58 years. Patients with ME/CFS were diagnosed by a clinician according to the Canadian Consensus^34^ and/or CDC-1994 (“Fukuda”)^35^ criteria, as determined by the responses on the Symptoms Assessment form to confirm case definition compliance and study eligibility.

Participants exclusion criteria included have (1) used drugs in the preceding three months known to alter immune function or taken anti-viral medications; (2) had any vaccinations in the preceding three months; (3) had a history of acute or chronic infectious diseases such as hepatitis B and C, tuberculosis, HIV (but not herpes virus or other retrovirus infection); (4) had another severe illness such as cancer, coronary heart disease, and uncontrolled diabetes; (5) had a severe mood disorder; (6) been pregnant or breastfeeding in the preceding 12 months; or present morbid obesity (BMI ≥ 40). Home visits were used for recruitment of patients with mobility restrictions (severely affected) while healthy subjects and mild/moderate patients were invited to a recruiting center for clinical assessment and blood sampling.

### Total RNA isolation

Plasma aliquots were thawed in a 37 °C bath for 1 min. Then, 200 µL were used for total RNA isolation using the miRNeasy Serum/Plasma Advanced Kit (#217,204, QIAGEN) in accordance with the operating manual. *C. elegans* miR-39 miRNA mimic (#219,610, QIAGEN) was added during the RNA extraction to monitor RNA recovery and reverse transcription efficiency. RNA aliquots of 20 µL were stored at -80 °C.

### First-strand cDNA synthesis and real-time PCR

miRCURY LNA RT Kit (#339,340, QIAGEN) was used to synthesize cDNA. 2 μL of RNA were used as a template for the reverse transcription reaction (60 min at 42 °C). Then, the samples were incubated for 5 min at 95ºC to heat inactivate the reverse transcriptase (peqSTAR 2X Gradient Thermocycler, Peqlab Biotechnologie GmbH). miRCURY SYBR Green PCR Kit (#339,346, QIAGEN) and miRCURY LNA miRNA PCR Assays (#339,306, QIAGEN) were used to evaluate the expression of hsa-miR-21-5p (YP00204230) (Gene ID: 406,991), hsa-miR-34a-5p (YP00204486) (Gene ID: 407,040), hsa-miR-92a-3p (YP00204258) (Gene ID: 407,048), hsa-miR-126-3p (YP00204227) (Gene ID: 406,913), and hsa-miR-200c-3p (YP00204482) (Gene ID: 406,985) relative to cel-miR-39-3p (YP00203952) by means of real-time PCR. 3 μL of cDNA (diluted 1:20) were used as a template for each reaction (final volume 10 μL). PCR cycling conditions included initial heat activation (2 min at 95 °C), 2 step cycling, denaturation (10 seg at 95 °C), combined annealing/extension (1 min at 56 °C), 40 cycles, and melting curve analysis (60–95 °C). The real-time PCR was performed in a 96-well thermocycler (StepOnePlus Real-Time PCR System, Applied Biosystems-Life technologies). cel-miR-39-3p served as the normalization control, and data were analyzed with Software v2.3 (Applied Biosystems-Life technologies) to obtain miRs relative expression scores. Cycle threshold (Ct) values of each miR were normalized to cel-miR-39-3p, and the 2^−ΔΔCt^ method was used to analyze the relative expression level of miR-21-5p, hsa-miR-34a-5p, hsa-miR-92a-3p, hsa-miR-126-3p, and hsa-miR-200c-3p in plasma samples from ME/CFSmm, ME/CFSsa patients and HCs.

### Statistical analysis of the UKMEB data derived from plasma

To compare basic characteristics between HC and patients with ME/CFS, t-tests for two independent groups were used for normally distributed data. The Mann–Whitney rank test for two independent groups was used otherwise. Given that CRP data had many values below the detection limit of 1, a linear regression based on truncated log-normal distribution was used to compare the different groups under analysis. In this analysis, it was fitted to two models, one including an intercept and a group variable and another one including an intercept only. These models were then compared with Wilks’ likelihood ratio test. *P* value < 0.05 indicated no differences between the three groups.

A pairwise correlation analysis between the five miR under analysis were conducted using Pearson’s correlation coefficients given that the respective log_10_-transformed data followed Normal distributions and the statistical relationship between any pair of miR was linear approximately. A similar analysis was also performed to assess the pairwise correlation between each miR and each clinical, blood and SF-36 variable but using Spearman’s correlation coefficient.

A principal component analysis on the miR data was used to assess the similarity of the study participants in terms of these variables. In this analysis we only used the first principal components because these components could account for more than 90% of the miR data variability. In addition, two linear discriminant analyses based on the miR data were performed to distinguish patients with ME/CFS from healthy controls and to distinguish severely affected patients from patients with mild or moderate symptoms. In general, linear discriminant analysis when applied to two-group case aims to find the best linear combination of a given set of variables that could distinguish two groups. The key output of this analysis is the probability of classifying each individual in the respective group given the estimated linear combination of the variables.

To detect differential expression of each miR between the three study groups, we estimated linear regression models defining the log10-transformed data of each miR as the outcome variable, and age, gender, body mass index, and two possible disease status variables (healthy vs ME/CFS and healthy vs ME/CFSmm vs ME/CFSsa) as the respective covariates. Three and four-way ANOVA were used to test the statistical significance of the covariates in the models including three and four covariates, respectively. Raw *p* values were then adjusted in order to ensure a global false discovery rate of 5% using the Benjamini–Hochberg procedure. Finally, the assumptions associated with linear regression modelling were confirmed by a residual analysis including normality assessment by Lilliefors and Shapiro–Wilk tests and different diagnostic plots (e.g., scatterplots of fitted values against standardized residuals).

The last step of the analysis was to estimate an empirical statistical power for detecting a group effect using the different estimated models including such effect as the true models for the respective data. With this purpose, we used the so-called parametric Bootstrap^36^ based on the following algorithm: (1) for each miR, simulate new values for each study participant according to the Normal distributions associated with the respective linear regression models estimated from the real data; (2) estimate the same models in the simulated data; (3) for each miR, calculate the *p* value to test the significance of the group effect as performed for the real data; (4) adjust the *p* values associated with each miR using the Benjamini–Hochberg procedure; (5) repeat the previous steps 1000 times in order to obtain 1000 data sets and to perform the respective analysis; and (6) estimate the power associated with each miR as the proportion of data sets which the group effect was statistically significant using adjusted *p* values as described for the analysis of the real data.

### Analysis of public microarray data derived from PBMCs

We re-analyzed a microarray data set from a previously published study^17^. In a nutshell, this data set consisted of 15 patients with ME/CFS and 29 HC matched for age and gender. Mean age of patients and HC was 43.8 and 44.76 years old, respectively. The proportion of males was 0.20 and 0.17 in patients and HCs, respectively. Patients with ME/CFS were diagnosed by the Fukuda 1994-CDC, but also fulfilled the 2003 Canadian Consensus Criteria. Additional information of the study participants can be found in the original reference^17^. The microarray data referred to log10-transformed intensities for around 385 miR as available in the Ambion Bioarray V1.0. We focused our analysis on data concerning the five miR of interest. Again, to detect differential expression of each miR between HC and patients with ME/CFS, we estimated linear regression models where the intensity data associated with each miR was the outcome variable while age, gender, and disease status (HC vs ME/CFS) were considered as the covariates. The diagnostics of the estimated regression model were conducted as described above for the analysis of plasma-derived data sample. A three-way ANOVA was used to test the statistical significance of the covariates in the estimated models. Again, raw *p* values were adjusted for multiple testing using the Benjamini–Hochberg procedure. The adjusted *p* values ensured a global false discovery rate of 5%. The whole data set is publicly available from the National Center for Biotechnology Information (NCBI) Geo data sets under the reference code GSE70371. The annotation file of the respective microarray can be found under the accession code GPL3444 in the same data repository. This file was used to identify each miR in the respective data set.

### Statistical software

All statistical analysis was performed in the R software version 4.0.2^37^ (https://www.r-project.org/). The following R packages were used: “tcensReg” to analyze the CRP data, “nortest” to test the normal distribution of quantitative variables and residuals of the linear regression analyses, “PerformanceAnalytics” to produce the correlation matrix shown in Fig. 2, and “MASS” to adjust raw *p *values for multiple testing. The R scripts used in this paper are freely available from NS upon request.

### Integrative network analysis

Potential miR-mRNA interactions were determined using miRTarBase 7.0 (http://mirtarbase.mbc.nctu.edu.tw/php/index.php), a database that estimates interactions based on experimentally validated assays (*e.g.* reporter gene assay, qPCR, and western blot). Protein–protein interactions (PPI) were also estimated using the GeneMANIA prediction server, which uses experimentally confirmed physical associations^38^. In order to analyze the PPIs topology network, a graphical representation was generated with Cytoscape software (version 3.7.2) using the Organic layout. The degree and betweenness centrality of proteins in the PPIs network were also calculated. The degree score (DS) corresponds to the count of how many interactions a node has in the whole network. On the other hand, betweenness centrality (BC) measures the frequency with which a node lies between the shortest communication path of all other possible pairs of nodes within a network, determining which are the key nodes for communication within the network. In this context a protein with a high DS and BC is likely an essential protein.

### Over-representation analysis

Over representation analysis (ORA), an approach to determine whether known biological functions or processes are enriched in an experimentally-derived gene list, was performed calculating a *p* value using hypergeometric distribution^39^. The list of miRs targets previously obtained using miRTarBase 7.0 were used as a target group. In parallel, a full list of genes obtained from the NCBI database was established as a background set of genes that function together in a known biological pathway. The analysis was performed using five random lists of genes obtained from miRTarBase 7.0 to ensure the biological processes are represented in the gene list more often than expected by chance. Random lists were generated using the random python module to determine if a significative gene ontology term could be obtained by chance.

## Results

### Study participant characteristics

The demographic and clinical characteristics of the participants are summarized in Table 1. The final age-matched cohort comprised 58 patients with ME/CFS divided into mild/moderate (mm) (ME/CFSmm: 28; females: 71%) and severely affected (sa) ones (ME/CFSsa: 30; females: 80%). Healthy controls included 29 participants (Females: 41%). Fatigue severity scores were higher both in ME/CFSmm and ME/CFSsa in relation to HC (*p* < 0.0001). As expected, clinical assessments related to pain and fatigue were higher in patients with ME/CFS compared to HC, with higher scores in ME/CFSsa comparing to ME/CFSmm (*p* < 0.0001). In contrast, waist circumference (*p* = 0.0124), body metabolic rate (*p* = 0.0044), and body muscle impedance (*p* = 0.0011) were lower, while body fat bioimpedance was higher (*p* = 0.0235) both in ME/CFSsa and ME/CFSmm patients compared to HC participants. Diastolic and systolic pressure, along with BMI were similar across the groups. Blood tests showed increased platelets (*p* = 0.0264), basophils (*p* = 0.0359), erythrocyte sedimentation rate (ESR) (*p* = 0.0110) and reduced levels of creatinine (*p* = 0.0045) and creatine phosphokinase (CPK) (*p* < 0.0001) both in ME/CFSmm and ME/CFSsa in relation to HC. The median obtained from the physical functioning scale variables (SF-36 questionnaire) such as physical functioning, role physical, bodily pain, general health, vitality, social functioning, role emotional, mental health, and both physical and mental component summary were significantly reduced in both ME/CFSsa and ME/CFSmm patients when compared to HC.

**Table 1:** Demographics of the study population based on sex, age, fatigue severity scale scores, clinical assessments, blood tests and SF-36 test. HC, participants recruited as healthy controls; ME/CFSmm, participants recruited as mild/moderate ME/CFS patients; ME/CFSsa, participants recruited as severely affected ME/CFS patients. Body mass index (BMI); erythrocyte sedimentation rate (ESR); creatine phosphokinase (CPK); C-reactive protein (CRP). *p values were obtained as follows: χ^2^ tests for categorical variables, Kruskal–Wallis test for non-normally distributed continuous variables and ANOVA test for normally distributed continuous variables. Normal distribution was checked by D’Agostino & Pearson test. Note that the median and interquantile for CRP were obtained from fitting a linear regression based on a truncated Lognormal distribution to the respective data.

Discrete variables	HC		ME/CFSmm		ME/CFSsa		*p value*
	(n)	(%)	(n)	(%)	(n)	(%)	
**Sex**							
Male	17	59	8	29	6	20	
Female	12	41	20	71	24	80	< 0.01

Continuous variables	HC		ME/CFSmm		ME/CFSsa		*p value*
	Median	IQR	Median	IQR	Median	IQR	
Age (years)	43	32–48	44	34–48	44	32–54	0.66
Fatigue severity scale scores	1.89	1.44–2.44	6.56	5.95–6.78	6.62	6.25–6.86	< 0.0001
**Clinical Assessments**							
Pulse oximetry (%)	99	98–99	98	97–98	99	98–99	0.03
Systolic pressure (mmHg)	126	111–131	124	110–136	120	108–129	0.48
Diastolic pressure (mmHg)	74	72–83	81	73–92	80	71–85	0.06
Height (cm)	171	168–175	168	162–178	165	159–177	0.31
Weight (kg)	80	66–86	74	63–86	63	54–81	0.06
BMI (kg/m^2^)	24	22–30	26	23–28	22	20–27	0.21
Waist circumference (cm)	90	78–102	91	87–100	79	72–90	0.01
Body fat	20	15–28	31	25–38	31	22–36	0.02
Body muscle	56	46–64	47	42–56	43	38–53	0.001
Body metabolic rate	1737	1469–1953	1509	1383–1685	1415	1197–1576	0.004
Pain analog scale	0.2	0.0–1.1	2.5	1.6–3.5	4.4	2.0–6.5	< 0.0001
Fatigue analog scale	0.8	0.0–1.7	5.2	3.6–6.9	7.1	6.0–8.2	< 0.0001
**Blood tests**							
Hemoglobin (g/L)	144	131–151	140	130–144	141	131–148	0.64
White blood cells (10^9^/L)	5.75	4.73–6.60	6.56	5.75–7.90	5.96	5.28–7.37	0.15
Platelets (10^9^/L)	227	203–280	274	231–336	264	221–297	0.03
Red blood cells (10^12^/L)	4.83	4.50–5.02	4.67	4.34–4.94	4.64	4.39–4.89	0.62
Hematocrit	0.430	0.400–0.454	0.427	0.388–0.449	0.422	0.395–0.439	0.89
Neutrophils (10^9^/L)	3.21	2.54–4.08	3.97	2.92	5.28	3.00–4.59	0.18
Lymphocytes (10^9^/L)	1.85	1.65–2.10	1.70	1.60–2.29	1.93	1.55–2.32	0.84
Monocytes (10^9^/L)	0.45	0.36–0.51	0.51	0.41–0.60	0.43	0.40–0.54	0.21
Eosinophils (10^9^/L)	0.12	0.08–0.17	0.13	0.07–0.22	0.14	0.08–0.19	0.93
Basophils (10^9^/L)	0.04	0.03–0.04	0.05	0.03–0.06	0.05	0.03–0.06	0.04
ESR (mm/h)	2.00	2–5	5	2–12	5	2–8	0.01
Creatinine (μmol/L)	86	75–93	75	68–79	69	59–76	0.01
CPK (U/L)	108	83–220	79	62–101	64	50–76	< 0.0001
CRP (CRP)	1.00	0.40–2.49	1.13	0.46–2.81	0.55	0.22–1.36	0.31
**SF-36 test**							
Physical functioning	57.5	57.1–57.5	32.7	28.8–37.5	21.2	19.3–25.0	< 0.0001
Role physical	57.2	54.4–57.2	28.0	22.4–33.6	21.2	21.2–25.7	< 0.0001
Bodily Pain	55.6	51.5–62.0	42.2	34.2–44.7	34.2	26.5–42.2	< 0.0001
General health	60.3	57.4–63.1	30.8	26.1–33.2	28.5	23.7–30.8	< 0.0001
Vitality	52.6	48.9–58.5	28.8	25.9–33.3	25.9	22.9–31.8	< 0.0001
Social functioning	57.3	57.3–57.3	27.2	19.7–32.3	17.2	17.2–22-3	< 0.0001
Role emotional	56.2	56.2–56.2	45.7	35.3–52.7	56.2	38.8–56.2	< 0.001
Mental health	54.8	48.3–58.7	40.4	36.5–48.3	48.3	40.4–53.5	< 0.001
Physical component summary	58.4	55–7-59.8	29.4	26.5–33.9	19.9	16.8–23.1	< 0.0001
Mental health component summary	54.2	47.8–57.1	41.8	34.4–45.0	45.9	43.6–50.4	< 0.0001

### Analysis of miR expression data from plasma samples

**Figure 1:**
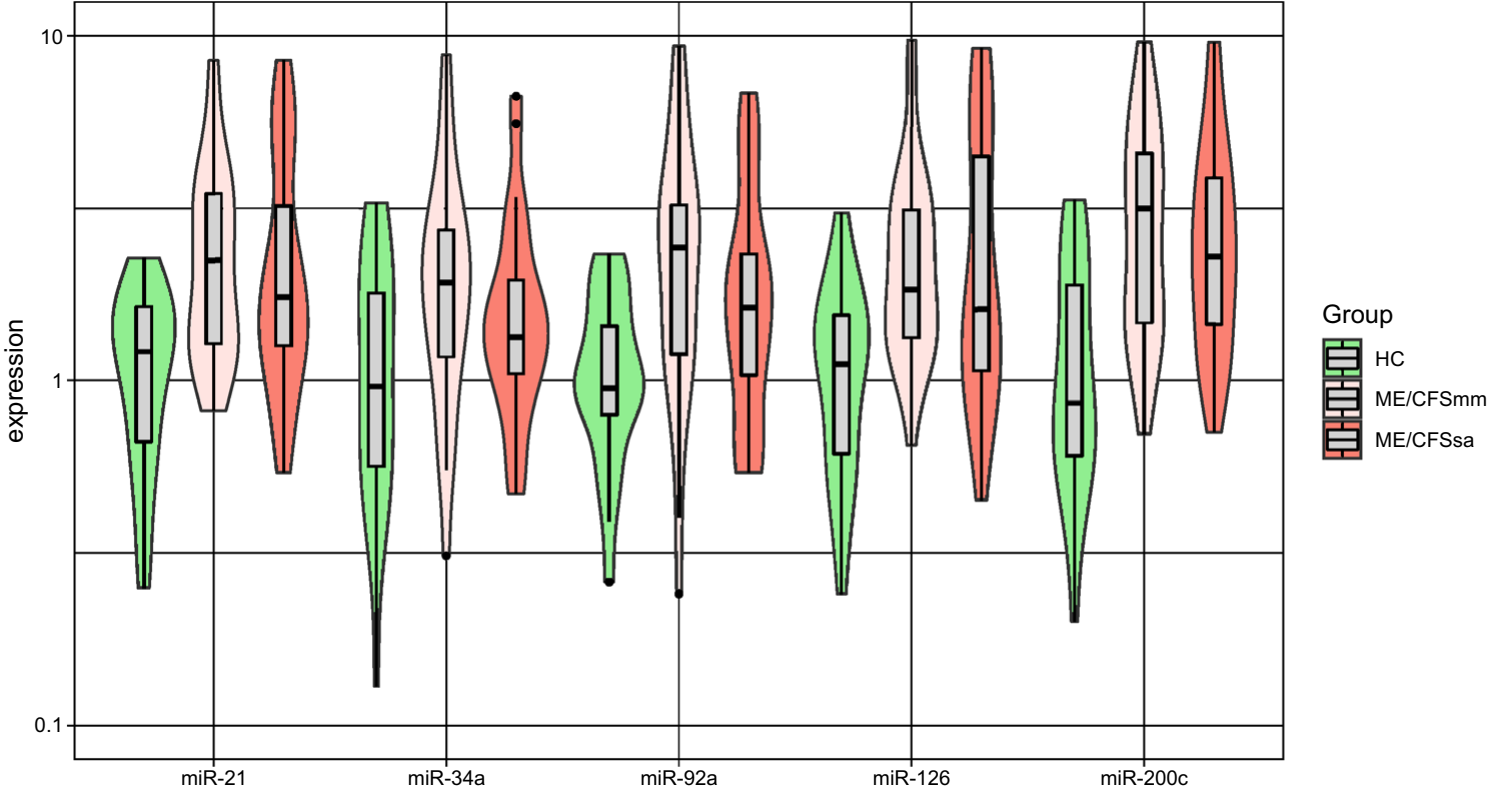
miR expression data derived from plasma samples. Violin plots summarizing the expression data from each miR in healthy controls (HC), mild/moderate (mm) and severely affected (sa) ME/CFS patients. Note that the violin plots represent the classical boxplot depicted inside the violin together with a density plot that gives the shape of the violin. The statistical analysis was performed in the R software version 4.0.2 (https://www.r-project.org/).

**Figure 2:**
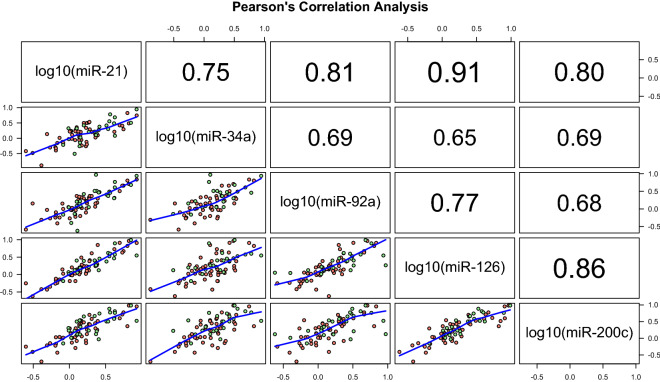
Correlation analysis between miRs expression data derived from plasma samples. Lower panel shows the scatterplots between the expression of a pair of miR taken in log10-scale where the blue line represents a lowess approximation of the respective relationship between x and y variables, and the dots in light green and salmon color represent healthy individuals and patients with ME/CFS, respectively. The values in upper panel are the Pearson´s correlation coefficient estimates for the corresponding data shown in lower panel. The statistical analysis was performed in the R software version 4.0.2 (https://www.r-project.org/).

**Figure 3:**
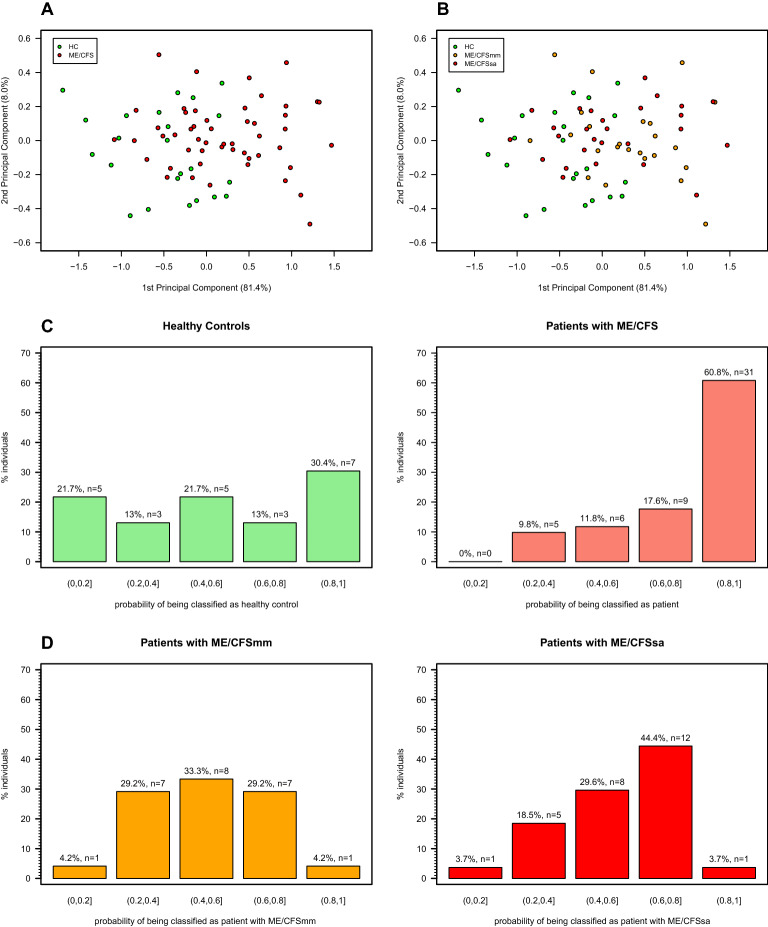
Principal component analysis and linear discriminant analysis derived from miR expression data in plasma. The scatterplots show the representation of each study participant in the first two principal components. The scatterplots (**A**) and (**B**) are the same but color-coded according to healthy controls (HC) versus patients with ME/CFS and according to healthy controls, mild/moderate (mm) and severely affected (sa) ME/CFS patients, respectively. The barplots (**C**) represent the frequency of HC and patients with ME/CFS and their corresponding classification probability of being classified as such according to a linear discriminant analysis distinguishing patients from controls specifically. The barplots (**D**) are similar to ones shown in (**C**), but for a linear discriminant analysis distinguishing mild/moderate patients from severely affected patients specifically. The statistical analysis was performed in the R software version 4.0.2 (https://www.r-project.org/).

We then used plasma samples from our cohort to analyze the relative abundance of the set of miRs reported to modulate the Sirt1/eNOS axis. We detected that the levels/quantities of miR-21, miR-34a, miR-92a, miR-126, and miR-200 were all increased in both ME/CFS groups compared to HC (Fig. 1), and that these miRs are highly and positively correlated with each other irrespective of the study group (Fig. 2). In line with this result, the first two principal components explained more than 90% of the variation in the data (Fig. 3). Interestingly, the principal component analysis showed that some patients with ME/CFS were clearly different from HC in the abundance of these miRs (Fig. 3A), but it seems not to be related to disease severity (Fig. 3B). To complement this visual interpretation of the principal components, linear discriminant analysis suggested that miR data could correctly classify 60.8% (n = 31) of patients with at least 80% probability (Fig. 3C). In contrast, only 30.4% (n = 7) of HC could be classified as such with the same minimum probability. When linear discriminant analysis was used to distinguish between ME/CFSsa and ME/CFSmm patients, the corresponding classification probabilities were in the vicinity of 50% for individuals from each disease group (Fig. 3D). In conclusion, miR expression data may be a supportive biomarker to discriminate between ME/CFS patients and HCs, but cannot predict disease severity.

**Figure 4:**
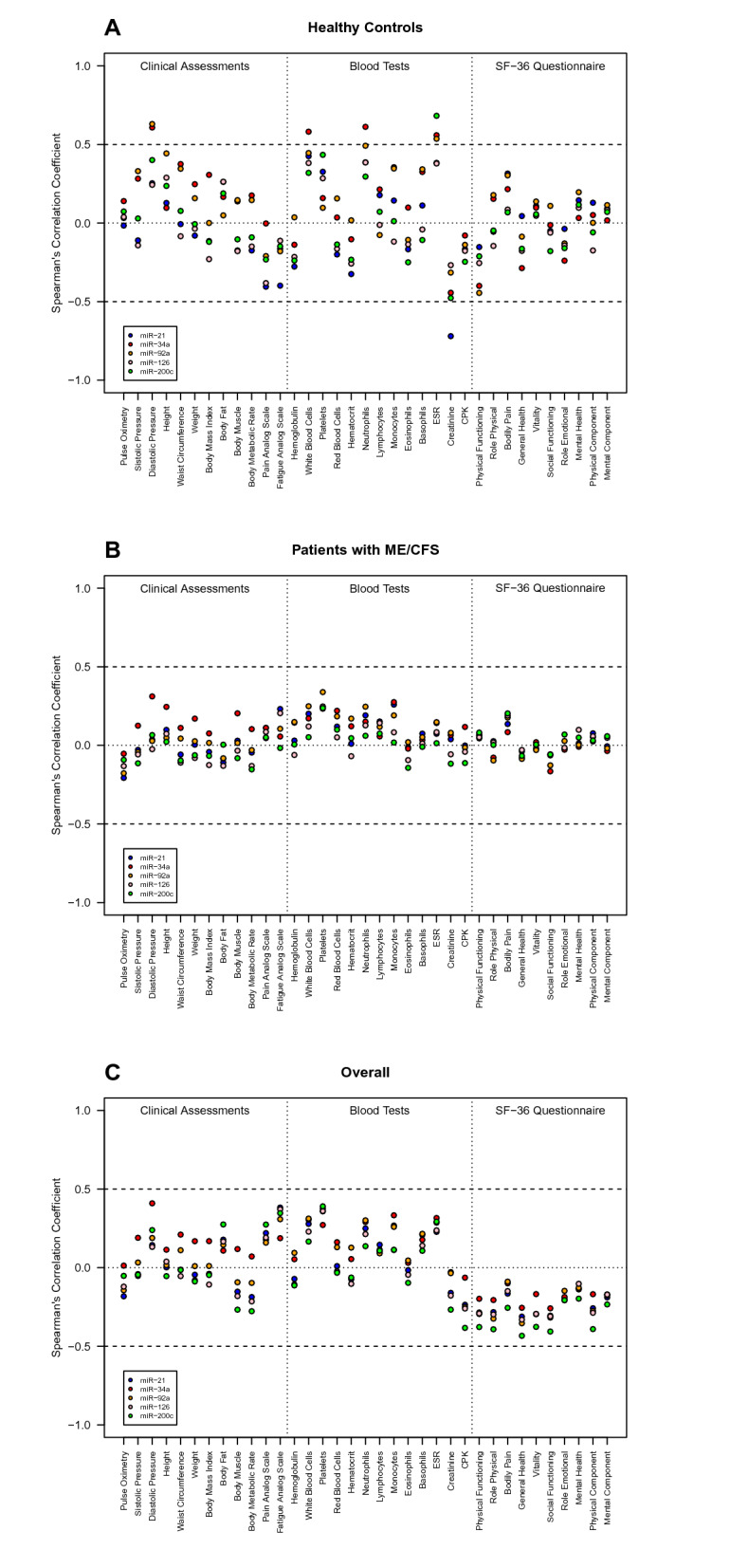
Correlation analysis between miR expression and clinical-related data. A pairwise correlation analysis between data of each miR and data of each clinical, blood and SF-36 variable was conducted using Spearman’s correlation coefficient. A. Correlation analysis using the data of healthy controls only. B. Correlation analysis using the data of patients with ME/CFS only. C. Correlation analysis using data irrespective of the study groups. The statistical analysis was performed in the R software version 4.0.2 (https://www.r-project.org/).

**Figure 5:**
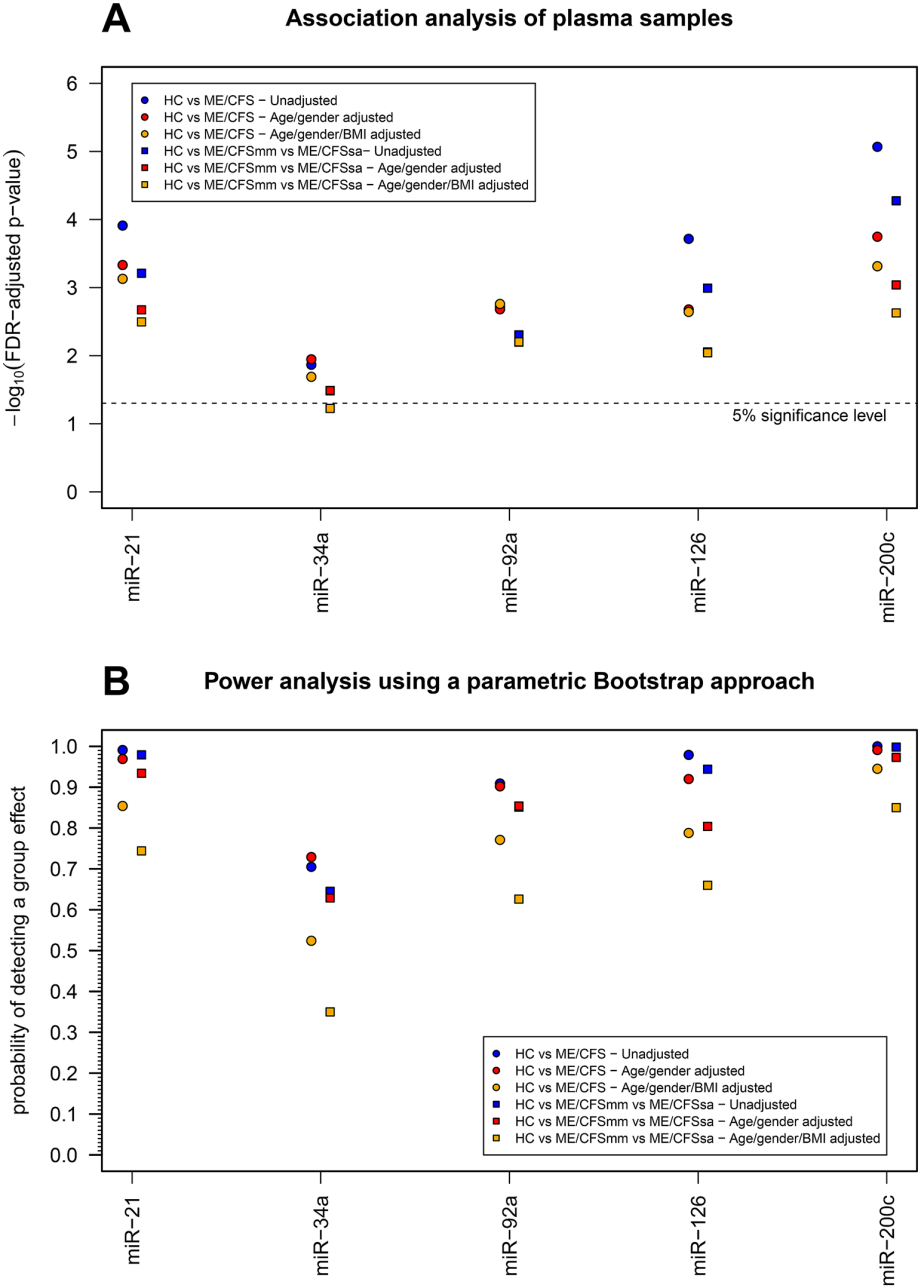
Association analysis between miR expression and study groups. (**A**) Association analysis adjusting for a global false discovery rate of 5% and controlling for age, gender, and BMI. Each dot represents the −log_10_(*p* value) of a specific statistical association test while the dashed line represents −log_10_(0.05) above which it was consider a statistically significant association (i.e., −log_10_(*p* value) > −log_10_(0.05)). *P* values were adjusted for a false discovery rate (FDR) of 5%. (**B**) Power analysis using a parametric Bootstrap approach under the assumption that the estimated models in A were the true ones for the data. In this analysis, the probability of detecting a group effect was estimated by the proportion of simulated data sets in which the group effect was statistically significant after adjusting for an FDR of 5%. The statistical analysis was performed in the R software version 4.0.2 (https://www.r-project.org/).

Spearman correlation coefficients were used to assess for an association between the increased Sirt1/eNOS-related miRs and each clinical parameter, in order to establish novel relationships that could help in better discriminating between patients and controls. In HC, the correlation coefficient involving ESR tends to be positive for miR-34, miR-92a and miR-200c. Diastolic pressure tends to be positive for miR-34, and miR-92a. The amount of white blood cells and neutrophils tends to be positively correlated with the level of miR-34a (Fig. 4A). The correlation estimates associated with ME/CFS patients (Fig. 4B) and all the participants from our cohort (HC and ME/CFS patients) (Fig. 4C) tends to be around zero for all miRs and clinical parameters, suggesting that the heterogeneity of ME/CFS patients might mask stronger associations related to the analyzed covariates. The association analysis shows the comparison between groups after adjusting by either age/gender, BMI or age/gender/BMI (Fig. 5A). All associations are statistically significant irrespective of their adjustment for possible confounders (Fig. 5A, Supplementary Table 1), highlighting the need to consider these analyses to obtain more reliable findings. The empirical power of detecting a group effect assuming the estimated models as the true ones for the data was in most cases higher than 80% in unadjusted analysis and in the analysis controlling for age and gender (Fig. 5B). This power tended to be lower in the analysis controlling for age, gender, and BMI. As expected, the lowest power was obtained for miR-34a which was already on the borderline of statistical significance after adjusting for multiple testing. In the worst-case scenario of this miR, the power of detecting a group effect was estimated at 35% for the analysis controlling age, gender, and BMI when using ME/CFSsa, ME/CFSmm and healthy controls as the group covariate.

### Analysis of microarray data from PBMCs

**Figure 6:**
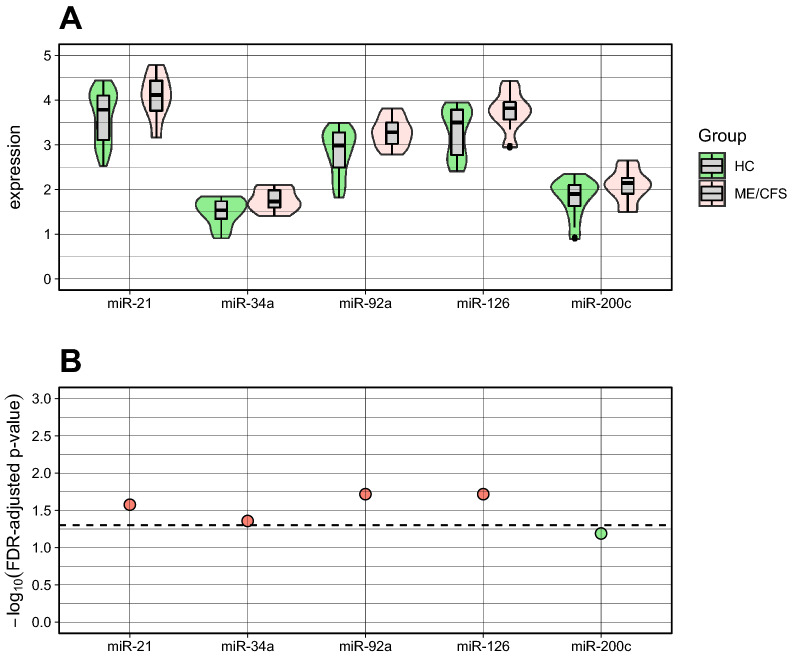
Analysis of publicly available microarray data derived from PBMCs. (**A**) Violin plots summarizing the expression data from each miR in PBMCs from healthy controls (HC) and patients with ME/CFS. (**B**) Association analysis adjusting for a FDR of 5% and controlling for age and gender where the dashed line represents -log_10_(0.05) and red dots represent the statistically significant associations between ME/CFS and the respective miR. Check legend of Fig. 5 for additional information about how to interpret this plot. The statistical analysis was performed in the R software version 4.0.2 (https://www.r-project.org/).

To provide additional evidence for the above findings on the UKMEB cohort of participants, we re-analyzed previously published microarray data from 15 patients with ME/CFS and 29 HC matched for age and gender^17^. We found that the five miRs under analysis were increased in patients with ME/CFS in comparison with HC (Fig. 6A, Supplementary Table 1). This increase was statistically significant for miR-21, miR-34a, miR-92a, and miR-126 after adjusting for multiple testing and controlling for possible confounding effects of age and gender (Fig. 6B, Supplementary Table 1). With respect to miR200c, the increase in expression was in the borderline of statistical significance (FDR-adjusted *p* value = 0.06). In conclusion, the five miRs are increased in both plasma (present data) and PBMCs^17^ from different cohorts, supporting their altered expression in ME/CFS.

### Bioinformatics analysis

**Figure 7:**
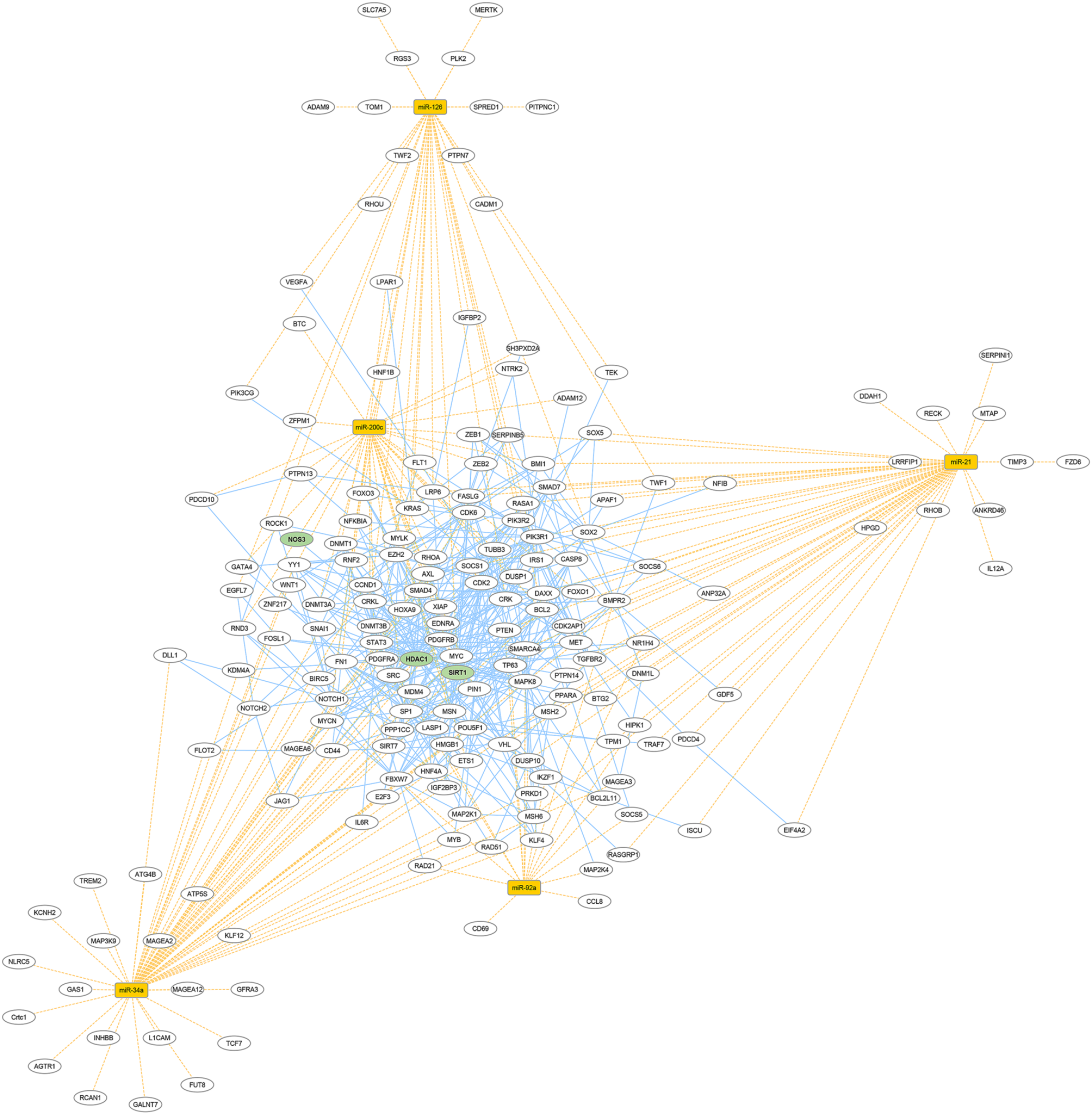
Visualization of miR-target interaction network. miR-21-5p, miR-34a-5p, miR-92a-3p, miR-126-3p, and miR-200c-3p are displayed in yellow squares, while white ovals represent their targets. Dotted yellow lines and solid blue light ones indicate miR-mRNA and protein–protein interaction, respectively. The graphical representation was generated with Cytoscape software version 3.7.2 using the Organic layout (https://cytoscape.org/).

**Figure 8:**
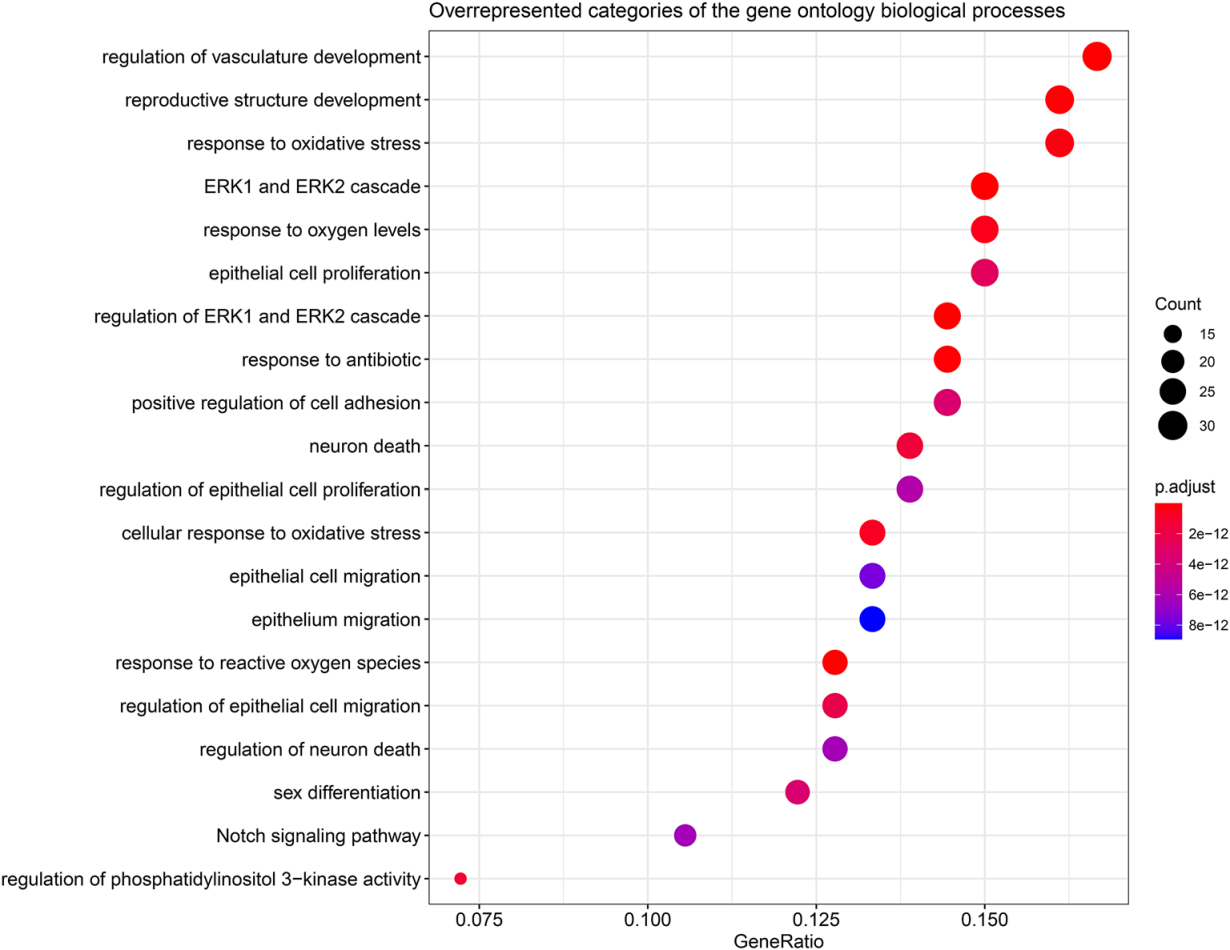
Biological processes related to miR-21-5p, miR-34a-5p, miR-92a-3p, miR-126-3p, and miR-200c-3p. Dot plot of functional enrichment analysis for the top 20 over-represented biological processes (BPs) related to our selected miRs. Dot sizes represent the number of genes (count) related to a particular BP. Gene Ratio is the number of genes found enriched in each category over the number of total genes associated to that BP. Dot colors represent the adjusted *p* values. Dot plots were generated in R software version 3.6.1 using the clusterProfiler package version 3.14.3 (https://bioconductor.org/packages/release/bioc/html/clusterProfiler.html).

Bioinformatics analyses were carried out to visualize targets and biological processes associated with the set of miRs. Based on miR-mRNA interaction analyses we observed 182 genes associated with miR-21 (n = 49), miR-34a (n = 61), miR-92a (n = 17), miR-126 (n = 33), and miR-200c (n = 38) (Fig. 7, Supplementary Table 2), indicating they are eventually regulated by more than one miR. After integration with protein–protein interaction data, we observed a cluster of 135 proteins with experimental evidence of physical interaction. Thus, our set of increased miRs is predicted to target highly interconnected proteins, suggesting a functional module (Fig. 7). Interestingly, histone deacetylase 1 (HDAC1) represented the most relevant node based on centrality measures (Fig. 7, Supplementary Table 2). Later, we identified several biological processes associated with miR-21-5p, miR-34a-5p, miR-92a-3p, miR-126-3p, and miR-200c-3p from a gene list validated experimentally. Functional pathway enrichment analyses showed that 5 out of 20 most over-represented biological processes are linked to regulation of vasculature development, followed by reproductive structure development, response to oxidative stress, ERK1/ERK2 cascade, and response to oxygen levels, respectively (Fig. 8). Additionally, 232 biological processes derived from the same analysis are also provided (Supplementary Table 3). Overall, the set of miRs selected for our study is mainly associated with endothelial function through regulation of highly interconnected proteins, where HDAC1 and Sirt1 are predicted as central players.

## Discussion

Despite ME/CFS has been studied for decades with significant progress achieved in the field, its etiology remains elusive. Accordingly, ME/CFS is diagnosed based on symptoms-related criteria which also leads to either undiagnosed or misclassified cases due to the heterogeneity of symptoms. Consequently, there is no single objective diagnostic or therapeutic biomarker, and current treatment is mainly focused on alleviating the complex symptomatology^1^. Therefore, there is an urgent need to identify reliable biomarkers for ME/CFS that allow a more precise diagnose and follow-up of its progression^40^. Thus, strategies to improve the reproducibility among studies such as the utilization of uniform clinical and research criteria, standardization of sample collection along with proper statistical analysis are certainly crucial to overcome this challenge.

We used plasma samples from healthy controls and age-matched mild/moderate and severely affected ME/CFS patients, obtained from the UKMEB^41^. In agreement with the stratification of groups in this study, all clinical parameters related with pain and fatigue were higher in ME/CFSsa than ME/CFSmm than HCs. In direct contrast, parameters associated with muscle mass (body muscle impedance) and muscle activity (creatinine and CPK) were lower in ME/CFSsa than ME/CFSmm than HCs, whereas body fat mass showed the opposite pattern, as expected due to the debilitating condition of the syndrome and the possibility of a gradual filling of the space of atrophic muscle tissue by fat tissue, as it occurs in other myopathies^42^, which, however, does not alter BMI despite a tendency towards a reduced body weight. As in other reports^43^, blood pressure and CRP were not different within our study population, in agreement with the immunological heterogeneity across ME/CFS cohorts. Furthermore, the non-specific marker of inflammation ESR, was found increased in ME/CFS patients compared to HCs, as well as reported in a different cohort from UKMEB^44^.

Notwithstanding the current knowledge that inflammation promotes ED in different diseases^6^, few studies have addressed endothelial function in vivo in patients with ME/CFS^7–9,43^. From a clinical perspective, Newton and colleagues^9^ reported ED in large and small arteries from ME/CFS patients using flow-mediated dilatation (FMD), linked to increased serum levels of CRP. Conversely, Moneghetti et al. neither report significant differences in CRP levels nor endothelial function assayed by peripheral arterial tonometry (EndoPAT) between ME/CFS patients still able to exercise and sedentary controls^43^. Scherbakov et al. also evaluated endothelial function by EndoPAT, showing ED associated with disease severity and immune symptoms in 51% of ME/CFS patients^7^. In line with these findings, Sørland et al. have recently reported ED in 40% of ME/CFS patients by using FMD^8^. Interestingly, our results show that ~ 61% of ME/CFS patients from our cohort might be classified as patients with at least 80% probability of suffering from ED, based on the increased set of miRs reported to regulate endothelial function via the Sirt1/eNOS axis. Although these findings also suggest that ED might be a trait observed in a subset (~ 40–60%) of ME/CFS patients, further studies focused on evaluating both endothelial function in vivo and this set of Sirt1/eNOS-related miRs in plasma from the same cohort might be of great value to support this assumption, offering a more sensitive approach to detect ED earlier.

The enzyme eNOS has a crucial role in endothelial function as a key mediator of vasodilatation by converting L-arginine into NO and L-citrulline. On the contrary, uncoupled eNOS and/or reduced eNOS activity induced by oxidative stress and inflammation triggers ED^6^. Sirt1 is another key modulator of endothelial homeostasis by activating eNOS to promote endothelium-dependent vasodilatation^11^, whose expression/activity is reduced during inflammation^10^. Over the last years, miRs have emerged as interesting candidates to identify biomarkers in ME/CFS^13–17^, since these noncoding RNAs play widespread roles in regulating gene expression^12^. Microarray analyses have identified several miRs differentially expressed in ME/CFS patients compared to HCs, also supporting by in silico analyses that inflammation-related processes and immune abnormalities play a role in its pathophysiology^14^. Then, we compared the expression of miR-21, miR-34a, miR-92a, miR-126, and miR-200c in plasma samples from ME/CFSmm and ME/CFSsa versus HCs, obtained from the UKMEB^41^.

We detected increased plasma levels of each miR in ME/CFS patients compared to HCs, independent of disease severity. In order to provide additional evidence, we also re-analyzed available microarray data in PBMCs from a different cohort of ME/CFS patients^17^. miR-21, miR-34a, miR-92a, and miR-126 were also found increased in patients with ME/CFS in comparison with HC, while miR-200c showed the same trend but at the borderline of statistical significance. Altogether, these findings not only support the fact that evaluating this set of miRs, reported to regulate the endothelial function via the Sirt1/eNOS axis, might be used to differentiate a subset of ME/CFS patients from HCs but also highlight the importance of available data sets to compare findings across studies.

Taking into account the immunological heterogeneity across patients during the course of the illness^2,3^, several studies have investigated immune patterns in ME/CFS, often with conflicting results^45^. Nevertheless, pro-inflammatory cytokines, such as TGF-β, TNF-α, IL-1β, and IL-6 seem to play a role related to illness duration and severity^2,3^. In agreement with a recent study carried out in PBMCs and extracellular vesicles from ME/CFSsa patients^15^, we found increased levels of miR-21 in plasma samples from both ME/CFSmm and ME/CFSsa patients compared to HCs. miR-21 is described to down-regulate the Sirt1/eNOS axis via TGF-β^20^ and TNF-α^28^ pathways in ECs and endothelial progenitor cells (EPCs), respectively. Increasing experimental and clinical data have also suggested that asymmetric dimethylarginine (ADMA), an endogenous eNOS inhibitor, promotes ED by reducing NO bioavailability^46^. Indeed, TNF-α-induced miR-21 up-regulation is linked to increased ADMA concentration and reduced eNOS activity in ECs^27^. L-citrulline, the by-product of NO synthesis from L-arginine by eNOS, ameliorated ADMA-induced ED by enhancing eNOS function and reducing oxidative stress^47^. Interestingly, L-citrulline was found significantly lower in plasma from ME/CFS patients compared to HCs^48^. Since the main source of L-citrulline is the gut, its circulating levels are used as an indicator of gut function^49^. Gut microbial dysbiosis has been proposed to be involved in ME/CFS^50^, and the degree of fatigue associated with the decrease of plasma L-citrulline in other pathological settings of dysbiosis^49^. Thus, increased plasma levels of ADMA^47,51^ and decreased plasma levels of L-citrulline^48^ may negatively affect NO production in ME/CFS patients. Additionally, miR-21 is thought to act as a positive but indirect regulator of Foxp3 expression in human regulatory CD4^+^ T cells (Tregs)^52^, a key cellular population of the adaptive immune system that controls autoimmunity and autoreactive T cells^53^. Accordingly, miR-21 ameliorated the clinical severity of experimental autoimmune encephalomyelitis in mice via a mechanism involving Tregs^53^. Therefore, it is possible to hypothesize that an overexpression of miR-21 could promote the expression of Foxp3 in naive CD4^+^ T cells and their conversion to the Treg pool. This putative Treg-cell conversion could explain the increase in the proportion of these cells in patients with ME/CFS observed in some studies^54^.

In line with this observation, miR-34 also regulates Tregs via NF-κB signaling^55^, a well-described pathway that induces inflammation by inhibiting Sirt1^10^. miR-34a also seems to play a key role in the regulation of endothelial cell inflammation by differentially modulating the antagonistic crosstalk between NF-κB and Sirt1^26^. Moreover, the negative regulation of miR-34a over the Sirt1/eNOS axis is associated with the premature senescence of ECs^25^. Interestingly, a randomized clinical study reported a negative correlation between miR-34 and Sirt1 expression in patients with coronary artery disease^29^. Thus, considering we found miR-34a increased in ME/CFS patients compared to HCs, further studies are certainly needed to elucidate its potential contribution to the immune and endothelial abnormalities observed in ME/CFS patients.

Our results also showed increased circulating miR-92a both in ME/CFSmm and ME/CFSsa patients, in agreement with a recent study using PBMCs^16^. In vitro, inhibition of miR-92a attenuates ED by decreasing the release of TNF-α and IL-6 by ECs^24^. In addition, miR-92a overexpression decreased eNOS activity and release of NO^23^, whereas its inhibition restored eNOS-mediated endothelial function by increasing Sirt1 expression in ECs^22^. In observational clinical studies, the circulating miR-92a positively correlated with microvascular coronary ED^30^. Moreover, miR-92a was inversely correlated with FMD and positively associated with IL-1β and CRP in serum from patients with coronary artery disease^22^.

In line with our findings, miR-126 was also found increased in extracellular vesicles from ME/CFSsa patients^15^. miR-126 is described to exert an endothelial protective role against hypoxia/reoxygenation-induced injury, oxidative stress, and TNF-α by activating the Sirt1/eNOS axis in ECs^21^. The circulating miR-126 was correlated to the improvement of endothelial function in male obese adolescents after exercise and diet control^31^. In addition, miR-126 reduces oxidative stress, IL-6, and TNF-α and activates both eNOS and vascular endothelial growth factor (VEFG) in ECs^21^. Recently, VEGF was found decreased in serum from ME/CFS patients^56^, an interesting finding considering that VEGF promotes survival and stability of ECs^57^. Altogether, higher plasma levels of miR-126 in ME/CFS patients might involve a compensatory mechanism against ED.

A large body of evidence suggests an autoimmune etiology in a subset of ME/CFS patients^7,58^. For instance, the M_3_ muscarinic acetylcholine receptor increases NO synthesis mediated by acetylcholine in ECs^59^, but autoantibodies against this receptor were found significantly higher in ME/CFS patients compared to HC^60^. Several autoantibodies, including those against the M_3_ muscarinic acetylcholine receptor, have been identified in people suffering from postural orthostatic tachycardia syndrome (POTS), characterized by tachycardia after moving from the supine to an upright position^61^, a condition also linked to ME/CFS^62^. Interestingly, miR-200c overexpression attenuates acetylcholine-induced endothelium-dependent relaxation in human renal arteries^19^. Thus, miR-200c might be related to acetylcholine-related ED. In this regard, circulating miR-200c was reported increased in atherosclerotic patients associated with reduced Sirt1/eNOS expression^32^. Additionally, miR-200c up-regulation induced by oxidative stress reduced Sirt1/eNOS axis activity^18^, causing apoptosis and senescence in ECs^63^.

Several bioinformatics analyses were also performed to visualize additional targets and biological processes associated with the set of miRs. Based on in silico analyses we validated that endothelial function-related signaling pathways are closely related to these miRs, including oxidative stress and oxygen regulation. Interestingly, HDAC1 was the most relevant node within our network built with miR-21, miR-34a, miR-92a, miR-126, and miR-200c. Histone deacetylases remove acetyl-lysine marks on proteins, especially in histones, leading to chromatin condensation and reduced levels of gene expression^64^. Changes in chromatin compaction through altered HDAC1^64^ along with other epigenetic alterations previously detected in ME/CFS patients^65^, might play also a role in this syndrome. Sirt1 deacetylates and activates HDAC1, but cell stress induces both Sirt1 degradation and HDAC1 acetylation, rendering HDAC1 less active^66^. Other HDAC isoforms have been reported increased in PBMCs from ME/CFS patients^67^. Notably, up-regulation of HDAC1 has been shown to reduce NO production by deacetylating eNOS in ECs in vitro^68^. Despite these findings might represent potential strategies for preventing ED via HDAC1, they should be interpreted with caution because both up-regulation of HDAC1^68^ and pharmacological inhibition of HDACs^69^ negatively affect endothelial function. Thus, we need further studies to confirm any alteration in HDAC1 amount and/or activity directly related to ED in our cohort. However, based on the mutual regulation between Sirt1 and HDAC1^66^, a better analysis of endothelial function should address HDAC1/Sirt1/eNOS axis as a novel platform to dissect the altered regulatory pathways leading to ED.

Noteworthy, we found similarities in the expression of our target miRs in plasma compared to PBMCs from another cohort^17^, but their origin is unknown. From a biological aspect, miRs are produced, released, and incorporated by different cell types, playing widespread roles in regulating gene expression throughout the body^70^. NF-κB is also involved in the modulation of a wide range of pleiotropic responses^10^. Thus, it is not surprising that this master transcriptional factor, well-known to modulate both immune and inflammatory responses^71^, might also participate in puzzling signaling pathways ranging from metabolic syndrome^72^ to depression^73^ in crosstalk with several miRs^74^, including miR-21^75^, miR-34a^55^, miR-92a^22^, and miR-126^76^. Based on the heterogeneous nature of ME/CFS^1^, we are aware that these increased miRs are not exclusively related to this syndrome, but they reflect an alteration, not related to severity, that might be further exploited to identify novel pathways with diagnostic and therapeutic value in ME/CFS. Since the ECs-related function varies from acute to chronic inflammation and even during the switch from innate to adaptive immunity^4^, further basic and clinical studies are certainly required to elucidate whether our studied miRs are involved in immune abnormalities in ME/CFS.

To our knowledge, this is the first report that collectively evaluates a set of circulating miRs associated with the Sirt1/eNOS axis, providing a basis for further studies to find ED-related biomarkers in ME/CFS. Overall, low-invasive detection of this set of miRs in plasma might help to identify ME/CFS patients, but their abundance per se cannot discriminate between ME/CFSsa and ME/CFSmm patients in this cohort. Our findings also support the hypothesis that endothelial homeostasis is an underestimated and partially addressed process which might play an important role in the complex pathophysiology of ME/CFS. Considering the pivotal function exerted by ECs and the fact that they are ubiquitous in tissues throughout the body, our study not only supports other reports associating ME/CFS with ED^7–9^ but also advances our understanding about potential new players not reported so far. In conclusion, we propose that a combination of clinical evaluation of endothelial function by FMD and/or EndoPAT, along with the detection of circulating Sirt1/eNOS-related miRs, might allow a more sensitive characterization of ED in a subset of ME/CFS patients for better stratification, which will certainly translate into improved treatment possibilities.

We are aware that these findings are not yet transferable into the clinical setting. Accordingly, observational studies with follow-up measurements in a bigger cohort are certainly needed to improve the power of the analysis and confirm our findings. In this line, we agree with Jason et al*.*^77^ that it is crucial to include minimum data in ME/CFS reports regarding patient characteristics, sampling, statistical methods, clinical and research assessments, as we intended to do in this report. Moreover, the use of more specific and restrictive case definitions represents a pivotal strategy to reduce misclassification bias^78^ and, as a consequence, improves the chances to identify reliable biomarkers in ME/CFS^40^.

For instance, despite the Fukuda case definition is the most used in ME/CFS research, it may misclassify individuals with major depression as ME/CFS patients^79^. In summary, including standardized diagnosis and research criteria into the study design will increase reproducibility/comparability among studies and positively impact ME/CFS patients’ health care.

## Supplementary Information

Supplementary Information 1.

Supplementary Information 2.

Supplementary Information 3.

Supplementary Information 4.

Supplementary Information 5.
