# Publisher Correction: Altered endothelial dysfunction-related miRs in plasma from ME/CFS patients

**DOI:** 10.1038/s41598-021-95087-3

**Published:** 2021-08-06

**Authors:** J. Blauensteiner, R. Bertinat, L. E. León, M. Riederer, N. Sepúlveda, F. Westermeier

**Affiliations:** 1 Institute of Biomedical Science, Department of HealthStudies, FH Joanneum University of Applied Sciences, Graz, Austria; 2 Centro de Microscopía Avanzada, CMA‑BIOBIO, Facultad de Ciencias Biológica, Universidad de Concepción, Concepción, Chile; 3 Instituto de Ciencias Biomédicas, Facultad de Ciencias de La Salud, Universidad Autónoma de Chile, Santiago, Chile; 4 Department of Infection Biology, Faculty of Infectious and Tropical Diseases, London School of Hygiene and Tropical Medicine, London, UK; 5 CEAUL - Centro de Estatística e Aplicações da Universidade de Lisboa, Lisbon, Portugal; 6 Institute of Medical Immunology, Charité‑Universitätsmedizin Berlin, Corporate Member of Freie Universität (FU) Berlin, Humboldt-Universität Zu Berlin and Berlin Institute of Health (BIH), Berlin, Germany; 7 Centro Integrativo de Biología y Química Aplicada (CIBQA), Universidad Bernardo O´Higgins, Santiago, Chile

Correction to: *Scientific Reports* 10.1038/s41598-021-89834-9, published online 19 May 2021

The original version of this Article contained errors.

Affiliation 5 was incorrectly given as ‘Centro de Estatística e Aplicações, Universidade de Lisboa, Lisbon, Portugal’. The correct affiliation is listed below.

‘CEAUL - Centro de Estatística e Aplicações da Universidade de Lisboa, Lisbon, Portugal’.

In Table 1, there is a repeated error in the superscripts where “kg/m^2^” was incorrectly given as “kg/m2”, “10^9^/L” was incorrectly given as “109/L” and “10^12^/L” was incorrectly given as “1012/L”.

Additionally, in the PDF part of the sentence “We detected increased plasma levels of each miR in ME/CFS patients compared to HCs, independent of disease severity.” was incorrectly placed next to Figure 5.

Part of the sentence “cohort of ME/CFS patients^17^.” was incorrectly placed next to Figure 6.

Lastly, part of the sentence “Our results also showed increased circulating miR-92a both in ME/CFSmm and ME/CFSsa patients, in agreement with a recent study using PBMCs^16^.” was incorrectly placed next to Figure 8.

The original Article has been corrected.

