# Supplementary material for: Altered endothelial dysfunction-related miRs in plasma from ME/CFS patients
Source: Sci Rep. 2021 May 19;11:10604. doi: 10.1038/s41598-021-89834-9 (PMC8134566; doi:10.1038/s41598-021-89834-9)
Supplement: Supplementary file 1 — Supplementary Information 1. [file 41598_2021_89834_MOESM1_ESM.pdf]

Supplementary Table 1

Coefficient estimates for the linear regression analysis for each miR in log10-scale using plasma and PBMC samples.

Samples	Coefficients	miR-21		miR-34a		miR-92a		miR-126		miR-200c	
		Estimate (SE)	P-value	Estimate (SE)	P-value	Estimate (SE)	P-value	Estimate (SE)	P-value	Estimate (SE)	P-value
Plasma[a]	Intercept	0.0669 (0.2212)	0.76	-0.2963 (0.2513)	0.24	0.0048 (0.2465)	0.98	0.0497 (0.2362)	0.83	-0.0356 (0.2408)	0.88
	Age	-0.0014 (0.0033)	0.66	0.0029 (0.0037)	0.43	0.0002 (0.0036)	0.95	0.0019 (0.0035)	0.59	0.0017 (0.0036)	0.63
	Gender (Female)	-0.0243 (0.0793)	0.76	-0.0934 (0.0895)	0.30	-0.1326 (0.0885)	0.14	0.0493 (0.0847)	0.56	0.0625 (0.0863)	0.47
	BMI	<0.0001 (0.0065)	0.99	0.0090 (0.0075)	0.23	0.0018 (0.0073)	0.81	-0.0058 (0.0070)	0.41	-0.0023 (0.0071)	0.74
	Group (ME/CFS)	0.3006 (0.0788)	<0.001	0.2100 (0.0884)	0.02	0.3006 (0.0878)	0.001	0.2731 (0.0841)	0.001	0.3554 (0.0858)	<0.0001
Plasma[b]	Intercept	0.0803 (0.2235)	0.72	-0.2847 (0.2535)	0.27	0.0211 (0.2485)	0.93	0.0608 (0.2388)	0.80	-0.0233 (0.2434)	0.92
	Age	-0.0015 (0.0033)	0.66	0.0030 (0.0037)	0.42	0.0002 (0.0036)	0.96	0.0019 (0.0035)	0.59	0.0017 (0.0036)	0.63
	Gender (Female)	-0.0181 (0.0804)	0.82	-0.0848 (0.0913)	0.36	-0.1248 (0.0895)	0.17	0.0544 (0.0859)	0.53	0.0681 (0.0876)	0.44
	BMI	-0.0007 (0.0067)	0.92	0.0083 (0.0076)	0.28	0.0010 (0.0074)	0.89	-0.0063 (0.0071)	0.38	-0.0029 (0.0072)	0.69
	Group (ME/CFSmm)	0.3224 (0.0875)	<0.001	0.2351 (0.0996)	0.02	0.3288 (0.0968)	<0.01	0.2911 (0.0935)	<0.01	0.3753 (0.0953)	<0.001
	Group (ME/CFSsa)	0.2731 (0.0920)	<0.01	0.1806 (0.1033)	0.09	0.2636 (0.1025)	0.01	0.2504 (0.0983)	0.01	0.3303 (0.1002)	0.001
PBMC	Intercept	4.4061 (0.4268)	<0.001	1.3549 (0.2938)	<0.001	2.9920 (0.3655)	<0.001	3.7154 (0.4053)	<0.001	2.0202 (0.3657)	<0.001
	Age	-0.0119 (0.0068)	0.09	0.0005 (0.0049)	0.91	-0.0021 (0.0058)	0.72	-0.0069 (0.0065)	0.29	-0.0044 (0.0060)	0.47
	Gender (Female)	-0.2829 (0.2265)	0.22	0.1586 (0.1395)	0.27	-0.0751 (0.1940)	0.70	-0.1501 (0.2151)	0.49	-0.0045 (0.1845)	0.98
	Group (ME/CFS)	0.4071 (0.1697)	0.02	0.2624 (0.1135)	0.03	0.4373 (0.1453)	<0.01	0.4409 (0.1611)	<0.01	0.2472 (0.1415)	0.09

[a] Linear regression with a group covariate distinguishing healthy controls (reference effect) from patients with ME/CFS

[b] Linear regression with a group covariate distinguishing healthy controls (reference) from mild/moderate patients with ME/CFS (ME/CFSmm) and severely-affected patients with ME/CFS (ME/CFSsa)
