# Supplementary material for: Altered endothelial dysfunction-related miRs in plasma from ME/CFS patients
Source: Sci Rep. 2021 May 19;11:10604. doi: 10.1038/s41598-021-89834-9 (PMC8134566; doi:10.1038/s41598-021-89834-9)
Supplement: Supplementary file 4 — Supplementary Information 4. [file 41598_2021_89834_MOESM4_ESM.pdf]

Supplementary Information

Altered endothelial dysfunction-related miRs in plasma from ME/CFS patients

Blauensteiner J, Bertinat R, León LE, Riederer M, Sepúlveda N, Westermeier F*
