# Supplementary material for: Altered endothelial dysfunction-related miRs in plasma from ME/CFS patients
Source: Sci Rep. 2021 May 19;11:10604. doi: 10.1038/s41598-021-89834-9 (PMC8134566; doi:10.1038/s41598-021-89834-9)
Supplement: Supplementary file 5 — Supplementary Information 5. [file 41598_2021_89834_MOESM5_ESM.docx]

# Supplementary Table 2

**Basic network statistics.** Betweenness centrality and degree are showed for each node in the network.

# Supplementary Table 3

1. **Overrepresentation analysis of gene ontology statistics.** ID: GO identifier; Description: Biological term; GeneRatio: Number of genes belonging to a category versus the total number of target genes; BgRatio: Ratio between all genes of the organism that belong to a category versus the total number of genes in the organism. Significance is represented by p.value and p.adjusted results. Also, the gene ID and counts for the target’s gene are shown.
